# Is the prefrontal cortex organized by supramodal or modality-specific sensory demands during adolescence?

**DOI:** 10.1016/j.dcn.2021.101006

**Published:** 2021-08-14

**Authors:** V. Sicard, D.D. Stephenson, A.B. Dodd, S. Pabbathi Reddy, C.R. Robertson-Benta, S.G. Ryman, F.M. Hanlon, N.A. Shaff, J.M. Ling, D.C. Hergert, K. Vakamudi, J. Hogeveen, A.R. Mayer

**Affiliations:** a The Mind Research Network/Lovelace Biomedical Research Institute, Albuquerque, NM, USA; b Department of Neurology, University of New Mexico, Albuquerque, NM, USA; c Department of Emergency Medicine, University of New Mexico Health Sciences Center, Albuquerque, NM, USA; d Department of Psychology, University of New Mexico, Albuquerque, NM, USA

**Keywords:** Multimodal, fMRI, Neurodevelopment, Attention, Supramodal, Visual dominance

## Abstract

Attention is inherently biased towards the visual modality during most multisensory scenarios in adults, but the developmental trajectory towards visual dominance has not been fully elucidated. More recent evidence in primates and adult humans suggests a modality-specific stratification of the prefrontal cortex. The current study therefore used functional magnetic resonance imaging (fMRI) to investigate the neuronal correlates of proactive (following cues) and reactive (following probes) cognitive control for simultaneous audio-visual stimulation in 67 healthy adolescents (13–18 years old). Behavioral results were only partially supportive of visual dominance in adolescents, with both reduced response times and accuracy during attend-visual relative to attend-auditory trials. Differential activation of medial and lateral prefrontal cortex for processing incongruent relative to congruent stimuli (reactive control) was also only observed during attend-visual trials. There was no evidence of modality-specific prefrontal cortex stratification during the active processing of multisensory stimuli or during separate functional connectivity analyses. Attention-related modulations were also greater within visual relative to auditory cortex, but were less robust than observed in previous adult studies. Collectively, current results suggest a continued transition towards visual dominance in adolescence, as well as limited modality-specific specialization of prefrontal cortex and attentional modulations of unisensory cortex.

## Highlights

• Evidence of an ongoing and potentially incomplete shift toward visual dominance observed in adolescents.
• Modality-specific stratifications of the prefrontal cortex were not present.
• Minimal functional evidence of proactive cognitive control and attention-related modulations was observed following cue presentation in adolescents.
• Current results suggest increased reliance on reactive control and ongoing transition toward sensory-specific information processing during adolescence rather than specialization.

## 1 Introduction

Cognitive control plays a fundamental role in continuously and flexibly adapting to changing environments (Diamond, 2013). The development of cognitive control reflects the continued refinement of an existing set of functions rather than the emergence of a new ability (Luna et al., 2015), and is therefore crucial to other cognitive domains (e.g., working memory, attention, and decision making; Diamond, 2013). To date, most developmental studies of cognitive control have used unisensory stimuli, which may not be reflective of the complex environmental multisensory demands encountered outside experimental settings.

According to the dual-mechanism framework, cognitive control operates via two distinct modes of proactive and reactive control (Braver, 2012; Maki-Marttunen et al., 2019), with reactive control maturing more rapidly from a developmental perspective (Munakata et al., 2012). Specifically, reactive control, or corrective actions to emergent and competing sensory or motor representations, is employed more frequently by younger children and older adults (Gonthier et al., 2019). In contrast, proactive control requires an individual to maintain goal-relevant information over sustained periods following an external or internal cue (Braver et al., 2007; Gonthier et al., 2016). It is believed to be mediated primarily by the sustained/increased activity of the cognitive control network (CCN). Proactive control is theorized to require additional metabolic resources (e.g., for glucose consumption, waste removal, neurotransmitter recycling), and places increased demands on working memory (Braver et al., 2009, 2007). Behavioral studies suggest that reliance on proactive or reactive cognitive control strategies changes from late childhood to early adulthood (Chatham et al., 2009; Gonthier et al., 2019; Lorsbach and Reimer, 2008, 2010; Munakata et al., 2012; Polizzotto et al., 2018), but the timing of the transition has not been well-defined.

In a multisensory environment, attention must sometimes be directed to a single sensory modality, which results in attention-related modulations (ARMs) within unisensory cortices (Hubel et al., 1959; Mayer et al., 2009; Wang et al., 2015). Auditory and visual cortex both show increased neuronal responses (i.e., up-regulation) when attention is focused on the respective sensory modality. A decreased neuronal response, posited to be indicative of inhibition, has also been observed within unisensory cortical areas corresponding to the unattended stimulus (Mayer et al., 2009). ARMs are believed to result from top-down feedback from the CCN, or potentially through direct innervation between unisensory cortex (Buffalo et al., 2010; Choi et al., 2018; Quak et al., 2015).

Sensory dominance depends on task context (Modality Appropriateness Hypothesis; Welch and Warren, 1980), perceived reliability of information, and stage of processing (Baier et al., 2006; Chen and Zhou, 2013; Ernst and Bulthoff, 2004; Mayer et al., 2009; Schmid et al., 2011). However, visual information dominates during most multisensory situations in adults with the exception of temporal processing (Visual Dominance theory or the Colavita effect; Colavita, 1974), with increased involvement of cortical regions when top-down attention is shifted away from visual streams (Mayer et al., 2017). In contrast, a recent meta-analysis concluded that the Colavita effect is absent in childhood (Hirst et al., 2018a). Additional behavioral studies suggest that infants and young children are biased towards auditory stimuli until approximately six to nine years of age, with the consolidation of visual dominance occurring in early adolescence (Barnhart et al., 2018; Chen et al., 2016; Hirst et al., 2018b; Nava and Pavani, 2013; Robinson and Sloutsky, 2004; Wille and Ebersbach, 2016). However, the majority of these studies only included youths up to age 12, with a notable absence of work conducted during adolescence.

It was traditionally assumed that there were different networks for attention to visual (dorsal frontoparietal cortex) relative to auditory (frontotemporal cortex) stimuli across major brain lobules (Braga et al., 2013, 2017), but that the prefrontal cortex (PFC) served in a supramodal capacity (Rahnev, 2017; Spagna et al., 2015, 2020; Wu et al., 2020a,b). However, non-human primate data (Barbas and Mesulam, 1985; Medalla and Barbas, 2014) and emerging evidence from adults (Braga et al., 2013, 2017; Mayer et al., 2017; Michalka et al., 2015; Noyce et al., 2017) suggest that activity in the lateral PFC may also be stratified dependent on the sensory modality during multisensory stimulation. Multisensory studies conducted in adult samples have reported the existence of interdigitating auditory and visual areas within lateral PFC that are stratified in a ventral-dorsal (Michalka et al., 2015; Noyce et al., 2017) or a rostral-caudal gradient (Mayer et al., 2017). However, whether these sensory-selective regions within the PFC are already present in adolescence or develop in early adulthood has yet to be determined.

The current study therefore extends prior work on multisensory cognitive control in adults (Mayer et al., 2017) to adolescents, purposefully altering the paradigm design to disambiguate neural activity in response to cues (theoretically measuring proactive control) and probes (theoretically measuring reactive control). Based on previous results (Mayer et al., 2017), it was hypothesized that the rostral lateral PFC would be specialized for auditory input, while the medial and caudal lateral PFC would demonstrate a preferential activation for visual input, yet also function in more of a supramodal capacity. Similarly, only the rostral lateral PFC was expected to exhibit modality-specific functional connectivity effects with auditory cortex (Mayer et al., 2017). We also predicted that ARMs would be more evident within the visual rather than auditory cortex, with increased activity within the posterior superior temporal sulcus (pSTS) observed for incongruent relative to congruent trials (Mayer et al., 2017; Mennigen et al., 2014).

## 2 Materials and methods

### 2.1 Participants

A jackknife analyses conducted in our healthy adult sample (Mayer et al., 2017) indicated that a minimum of 55 participants was required to reliably detect PFC stratification during multisensory tasks. Seventy-four healthy adolescents (13–18 years) were therefore included in the current study, with recruitment occurring from the local community using flyers and word of mouth. Exclusion criteria for the participants included 1) a history of a) neurological diagnosis, b) previous traumatic brain injury with >5 min loss of consciousness, c) developmental disorder (autism spectrum disorder or intellectual disability), d) any psychiatric disorders other than adjustment disorder, e) attention-deficit/hyperactive disorder, f) learning disorder, 2) non-fluency in English, or 3) substance abuse/dependence. To confirm drug abstinence, urine-based drug screens were conducted for all participants. Informed consent and assent, according to institutional guidelines at the University Of New Mexico School of Medicine, was provided by the parent or by the participants themselves if they were 18 years old.

Data acquisition errors occurred for three participants and an abnormal finding was present on the radiological report for one participant, resulting in study exclusion. One participant demonstrated extreme voxelwise activation (i.e., 3 standard deviations above the mean) in >5 % of voxels across all task conditions relative to the remainder of the sample and was removed prior to analyses. Two participants were excluded for poor accuracy (<64 % on two or more conditions) based on a binomial distribution. No participants were excluded as reaction time outliers (>3*interquartile range), nor were any participants excluded as motion outliers (mean framewise displacement [FD] >3*interquartile range). The final sample for task-based analyses therefore included 67 participants (31 females; 61 right-handed; mean age = 15.60 ± 1.68 years). FreeSurfer segmentation of the brain failed for one participant, resulting in a reduced sample for connectivity analyses (N = 66).

### 2.2 fMRI task paradigm

**Fig. 1:**
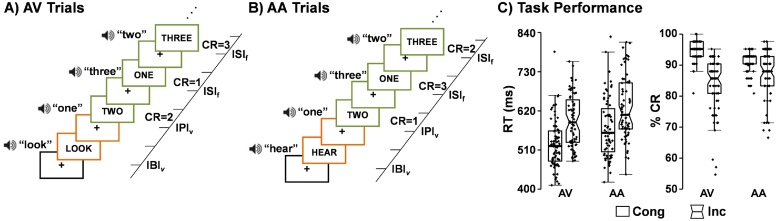
Diagrammatic representation of the task and behavioral results. Participants attended to target stimuli (numbers: one, two, or three) in either the visual (attend-visual: AV; Panel A) or auditory (attend-auditory: AA; Panel B) modality while ignoring incongruent (Inc) or congruent (Cong) distractor stimuli in the opposite modality. Each trial was separated into distinct cue (multisensory cue words “HEAR” or “LOOK”; orange colored boxes) and target (written Arabic numbers; green colored boxes) phases with corresponding hemodynamic response functions. This was accomplished by having variable inter-phase intervals (IPI_v_) between the cue and the first numeric target, as well as variable inter-block intervals (IBI*_v_*; black colored box). The inter-stimulus interval (ISI_f_) between numeric targets within each block was fixed. Correct responses (CR) are indicated on the right side of panels. Box-and-scatter plots (Panel C) depict median reaction times (RT) and accuracy for both AV and AA conditions for congruent (un-notched boxes) and incongruent (notched boxes) trials.

The current task was similar to previous studies (Mayer et al., 2017) with a few key modifications. Briefly, a multisensory cue (audiovisual; 300 ms duration) was presented at the beginning of each block (Fig. 1A and B) to determine the modality for selective attention (“HEAR” = attend-auditory; “LOOK” = attend-visual) or for the planned inhibition of upcoming motor responses (“NONE” = proactive response inhibition). The participants were instructed to withhold their response following the “NONE” cues. The NONE trials were modeled separately from the attend-auditory and attend-visual conditions at the single-subject level, but were not included in group-level analyses in the current study. Data on “NONE” trials in a subset of participants has been reported in a previous publication (Mayer et al., 2019b).

For attend-auditory and attend-visual conditions, the multisensory probes were either congruent (i.e., matching auditory/visual number) or incongruent (i.e., different auditory/visual number). In contrast to previous studies, a variable (2,460−3,380 ms) delay separated the presentation of multisensory numeric probes (words = “ONE”, “TWO”, or “THREE”; 300 ms duration) from cues. Therefore, the hemodynamic response function (HRF) could be individually and separately modeled for both the cue (theoretical proactive control) and probe (theoretical reactive control) phase. Probes occurred at a frequency of 0.66 Hz (6 trials per each 7.8 s block). Participants completed 7 blocks of each of the 5 conditions (i.e., none incongruent, attend-visual congruent, attend-visual incongruent, attend-auditory congruent, attend-auditory incongruent) for a total of 42 trials of each condition. Inter-block intervals were also jittered (3,700−5,540 ms) to decrease temporal expectations and minimize the non-linear summing of HRFs across cues and probes (Glover, 1999). The task design resulted in a non-singular/invertible matrix with only moderate collinearity.

Participants responded with a right-handed button press to one of three buttons corresponding to the target stimulus in the attended modality (probe modality) while ignoring simultaneously presented numbers in the opposite sensory modality. All multisensory stimuli were presented foveally and binaurally via headphones (head-centered). Participants were asked to maintain constant head and eye positioning (visual fixation on a centrally presented cross). All participants practiced the task outside of the MRI before the scan (one block of each condition, 6 trials per block; multiple practices possible). For the resting-state scan, participants were instructed to stare at a centrally presented white fixation cross on a black background for approximately 5 min.

Response time and accuracy data were modeled with either normal or gamma distributions as appropriate using 2 × 2 [Modality (Attend-auditory vs. Attend-visual) × Congruency (Congruent vs. Incongruent)] generalized estimating equations. Omnibus analyses were Bonferroni-corrected based on the number of comparisons (i.e., reaction time and accuracy; 0.05/2 = 0.025).

### 2.3 MR imaging

MRI data were collected on a 3 T Siemens Tim Trio scanner with a 32-channel head coil. Data collection included a high-resolution MPRAGE T_1_-weighted (1.0 × 1.0 × 1.0 mm) sequence, T_2_-weighted (1.1 × 1.1 × 1.5 mm) sequence, susceptibility-weighted images (1.0 × 1.0 × 1.5 mm), and fluid-attenuated inversion recovery images (0.8 × 0.8 × 3.0 mm). Collected images were reviewed by a blinded, board-certified radiologist. Task (2 runs) and connectivity (1 run) data were acquired utilizing a single-shot, gradient-echo echoplanar pulse sequence with 56 interleaved slices acquired for whole-brain coverage (3.02 × 3.02 × 3.00 mm) using multiband imaging to achieve high temporal sampling (TR = 460 ms) of the HRF. Initial images from task and resting-state runs were excluded to account for T_1_ equilibrium effects based on default calculations for multiband CMRR sequences, leaving 1,212 and 649 images, respectively. A reference image (multiband factor = 1) was also acquired to facilitate registration to T_1_-weighted data. Two spin-echo field mapping sequences (3.02 × 3.02 × 3.00 mm) with reversed-phase encoding directions (A→P; P→A) were generated to correct for susceptibility-related distortion.

### 2.4 Image processing and statistical analyses

Task and resting-state data were assessed for anomalous values and replaced using AFNI’s despiking tool (Cox, 1996). Images were then temporally interpolated to the first slice and spatially registered to a reference image in two- and three-dimensional space using AFNI software suite tools to reduce the effects of head motion. Images were corrected for susceptibility distortions using FSL’s Topup algorithm (Andersson et al., 2003; Smith et al., 2004), converted to standard stereotaxic space (Talairach and Tournoux, 1988) using a non-linear transformation (AFNI 3dQwarp) and spatially blurred (6-mm Gaussian filter). For task data, finite impulse response deconvolution was used to generate a single HRF individually for cue (14.26 s post-cue onset) and probe (20.70 s post-probe onset) phases. Nuisance regressors included 6 motion parameters (3 rotational and 3 translational) and their derivatives, error trials, and a second-order polynomial to reduce hardware-related artifacts (Mayer et al., 2019a). Resulting beta coefficients were then separately summed within individual HRF periods to obtain estimates of peak (2.30–5.98 s) and inhibitory (6.90–11.04 s) activity during the theoretical proactive cue phase. Beta coefficients were only summed within the probe HRF to obtain an estimate of peak (3.68–12.88 s) activity during theoretical reactive control conditions. These time windows were selected prior to analyses based on averaged (all participants across all trials) HRF data from motor, sensory, and PFC to avoid bias.

Resting-state functional connectivity maps were calculated by regressing motion parameters and their first-order derivatives, as well as the estimates of physiological noise from white matter and cerebral spinal fluid, followed by the application of a bandpass filter (0.01−0.1 Hz) to the data. Seed-based regions of interest (ROIs) for connectivity maps were defined within FreeSurfer (Version 5.3.0) using the Desikan-Killiany atlas (Desikan et al., 2006) for primary/secondary auditory and visual cortices (see Supplementary Materials for further details). Consistent with previous studies (Mayer et al., 2017), additional connectivity analyses were also conducted on empirically-derived seeds for those unisensory cortical regions that exhibited high activation in response to the relevant auditory and visual probes to verify results from the Desikan-Killiany atlas-based labels (see Supplementary Fig. S1 for a comparison of findings).

Two whole-brain one-way (Auditory cues vs. Visual cues) ANCOVAs with mean FD as a covariate examined for differences during peak and inhibitory cue phases. In addition, whole-brain 2 × 2 [Modality (Auditory cues vs. Visual cues) × Congruency (Congruent vs. Incongruent)] ANCOVAs with mean FD as a covariate examined differences in modality-specific activity during the probe phase. All whole-brain results within each modality were corrected for family-wise error using statistical (*p* < 0.001) and minimum volume thresholds (task-based analyses: 575 μL; connectivity analyses: 1067 μL) based on 10,000 Monte Carlo simulations and spherical autocorrelation estimates (Cox et al., 2017). All analyses were performed with and without age as a covariate in the model. As functional results were nearly identical, the more parsimonious model is presented in main results, whereas age covariate results are presented in Supplemental materials. The size of activated areas is reported in μL (1 native voxel = 27 μL).

## 3 Results

### 3.1 Behavioral data

Results are presented in Fig. 1C. The analysis for reaction time indicated a main effect of modality (Wald-χ^2^ = 79.82; *p* < 0.001), with a longer response time for attend-auditory (599.98±79.45 ms; data presented as mean ± standard deviation) relative to attend-visual trials (560.12±66.22 ms). Further, a main effect of congruency was observed (Wald-χ^2^ = 135.30; *p* < 0.001), with a longer response time for incongruent (610.52±73.26 ms) relative to congruent trials (549.58±73.91 ms). The Modality × Congruency interaction was not significant (Wald‐χ^2^ = 2.65; *p* = 0.10).

The analysis for accuracy indicated a significant Modality × Congruency interaction (Wald‐χ^2^ = 30.21; *p* < 0.001). For the congruent condition, accuracy on the attend-visual trials (94.64±3.19 %) was significantly higher relative to the attend-auditory trials (91.08±3.19 %), whereas for the incongruent condition accuracy was significantly higher on the attend-auditory trials (87.51±7.45 %) relative to attend-visual trials (83.49±8.58 %).

### 3.2 Functional task results

#### 3.2.1 Activity during cues

**Fig. 2:**
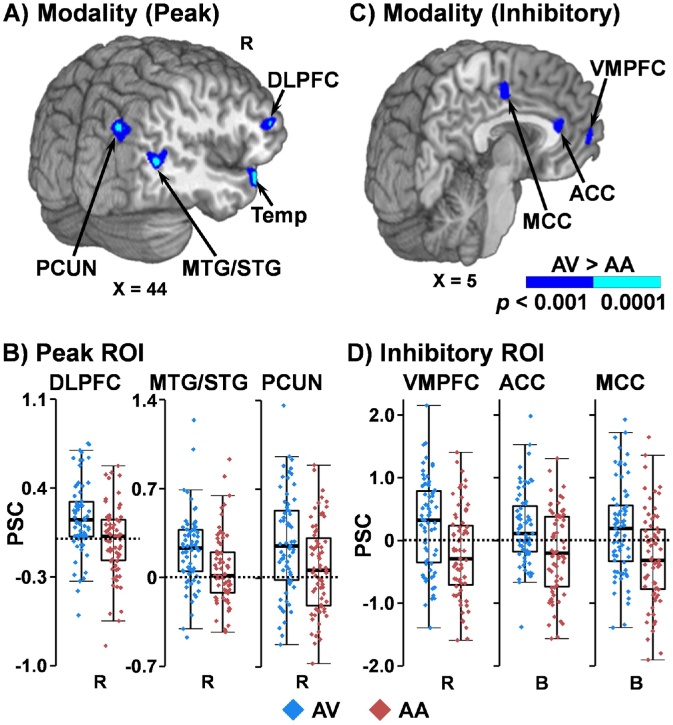
Cue-related activity and percent signal change (PSC) in selected regions of interest (ROI). During the peak phase (Panel A), increased activation (blue: p < .001; cyan: p < .0001) within the right dorsolateral prefrontal cortex (DLPFC), temporal pole (Temp), middle and superior temporal gyrus (MTG/STG), and precuneus (PCUN) was observed for the attend-visual (AV) relative to attend-auditory (AA) cues. During the inhibitory phase (Panel C), greater deactivation was observed for AA cues (blue: p < 0.001; cyan: p < 0.0001) within the ventromedial prefrontal cortex (VMPFC), anterior (ACC) and middle (MCC) cingulate cortex relative to baseline. Locations of the sagittal (X) slices are given according to the Talairach atlas. Panels B and D (R = right; B = bilateral) display box-and-scatter plots of the PSC (blue: AV; red: AA).

Results from whole-brain one-way ANCOVAs (attend-auditory cues vs. attend-visual cues) with mean FD as a covariate indicated increased activity during the peak phase in the right dorsolateral PFC (DLPFC) and right temporal pole, as well as aspects of the dorsal and ventral processing stream including the right middle/superior temporal gyrus (MTG/STG) and right precuneus extending to right superior occipital gyrus for attend-visual cues (Fig. 2A and B; Table 1). During the inhibitory phase (Fig. 2C and D), decreased activity (i.e., below baseline) was observed for attend-auditory cues in the right ventromedial PFC, as well as in the bilateral anterior and middle cingulate cortex.

**Table 1:** Main effect of modality during cue phase of the multisensory cognitive control task.

Area	BAs	CoM (X,Y,Z)	Volume	Detailed pattern
**Peak phase**				
Right DLPFC	10/46	35, 40, 11	1601 μL	AV > AA
Right temporal pole	38	48, 19, −16	807 μL	
Right MTG/STG	19/39	44, −57, 11	887 μL	
Right precuneus/superior occipital gyrus	19	32, −69, 34	2351μL	
**Inhibitory phase**				
Right VMPFC	10/32	9, 48, −1	614μL	AV > AA (deactivation)
Bilateral ACC	24/32	1, 32, 13	668μL	
Bilateral middle cingulate cortex	24	4, −12, 42	837μL	

Note: AA = attend-auditory; ACC = anterior cingulate gyrus; AV = attend-visual; BAs = Brodmann Areas; CoM = Center of Mass; DLPFC = dorsolateral prefrontal cortex; MTG/STG = middle/superior temporal gyrus; VMPFC = ventromedial prefrontal cortex.

#### 3.2.2 Activity during probes

**Fig. 3:**
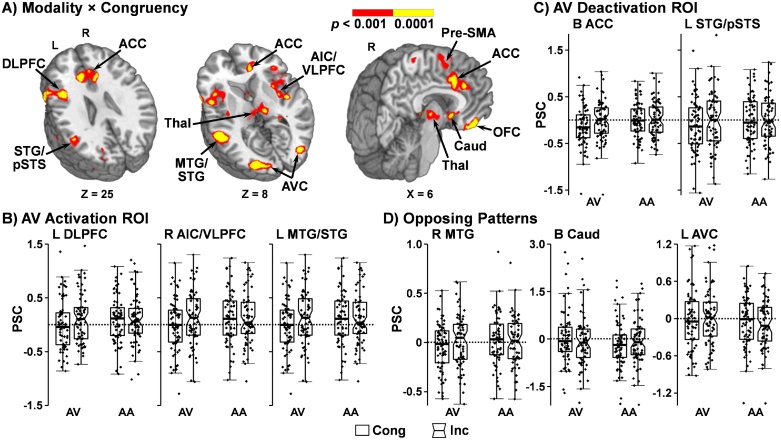
Modality × Congruency interaction and percent signal change (PSC) during probe-related activity in selected regions of interest (ROI). Regions that exhibited a significant Modality × Congruency interaction (red: p < 0.001; yellow: p < 0.0001) are depicted in Panel A. Locations of the sagittal (X) and axial (Z) slices are given according to the Talairach atlas. The dorsolateral prefrontal cortex (DLPFC), pre-supplementary motor area (Pre-SMA), anterior insular cortex/ventrolateral prefrontal cortex (AIC/VLPFC) and middle/superior temporal gyrus (MTG/STG) and bilateral thalamus (Thal) showed greater activation for the incongruent (Inc; notched boxes) relative to congruent (Cong; un-notched boxes) condition during attend-visual (AV) trials. Box-and-scatter plots of the PSC from selected ROI (R = right; L = left; B = bilateral) are depicted in Panel B. The anterior cingulate cortex (ACC) and the STG/posterior superior temporal sulcus (pSTS) showed deactivation relative to baseline during AV but not attend-auditory (AA) trials during the congruent condition (box-and-scatter plots in Panel C). Patterns of opposing activity (i.e., greater activation/deactivation for the incongruent and congruent conditions as a function of attended modality; Panel D) were observed within the orbitofrontal cortex (OFC), MTG, caudate (Caud) and associative visual cortex (AVC).

A 2 × 2 whole-brain ANCOVA (Modality [Attend-auditory vs. Attend-visual] × Congruency [Congruent vs Incongruent]) with mean FD as a covariate indicated several regions exhibiting a Modality × Congruency interaction that followed three general patterns (Table 2; Fig. 3A). First, greater activation for the incongruent relative to congruent condition for attend-visual trials, with no difference during the attend-auditory trials within the left DLPFC, bilateral pre-supplementary motor area (pre-SMA), right and left anterior insular cortex/ventrolateral PFC, left MTG/STG, right and left fusiform gyrus, and bilateral thalamus (Fig. 3B). Second, a greater deactivation (Fig. 3C) was observed in the congruent condition during the attend-visual trials, with no difference during attend-auditory trials within the bilateral anterior cingulate gyrus (ACC), and the left STG extending to the pSTS (evidence of ARMs). In the third pattern, opposing patterns of activity were observed for attend-visual and attend-auditory trials within the bilateral orbitofrontal cortex, right MTG extending into the internal capsule, and right primary sensorimotor cortex (Fig. 3D). Similarly, the bilateral caudate, the left middle frontal gyrus extending into anterior corona radiata, and the right and left associative visual cortex (evidence of ARMs) exhibited opposing patterns of activation and deactivation as a function of attended modality.

**Table 2:** Modality x Congruency interaction during probe phase of the multisensory cognitive control task.

Area	BAs	CoM (X,Y,Z)	Volume	Detailed pattern
Left DLPFC	9/45/46	−44, 16, 23	4862 μL	AV: I > C; AA: I ≈ C
Bilateral pre-SMA	6/8	0, 8, 53	3123 μL	
Bilateral AIC/VLPFC	R: 13/47	R: 40, 9, 1	R:6960 μL	
	L: 13/47	L: −39, 10, 0	L:6480 μL	
Left MTG/STG	21/22	−53, −34, 5	3092 μL	
Bilateral fusiform gyrus	–	R: 39, −55, −9	R: 644 μL	
		L: −37, −56, −8	L: 2084 μL	
Bilateral thalamus	–	4, −18, 5	2174 μL	
Bilateral ACC	32	3, 23, 26	7564 μL	AV: I > C (deactivation); AA: I ≈ C
Left STG/pSTS	22/40	−51, −48, 23	1505 μL	
Bilateral OFC	11	2, 35, −17	1399 μL	Opposing patterns of activity
Right MTG/internal capsule	–	39, −34, 1	1559 μL	
Bilateral caudate	25	3, 8, −3	870 μL	
Right primary sensorimotor cortex	3/4	19, −35, 60	2376 μL	
Left MFG/anterior corona radiata	–	−18, 24, −8	581 μL	
Bilateral associative visual cortex	R: 19	R: 30, −80, 11	R: 1306 μL	
	L: 19	L: −25, −82, 11	L: 3448 μL	

Note: AA = attend-auditory; ACC = anterior cingulate gyrus; AIC/VLPFC = anterior insular cortex/ventrolateral prefrontal cortex; AV = attend-visual; BAs = Brodmann Areas; C = congruent; CoM = Center of Mass; DLPFC = dorsolateral prefrontal cortex; I = incongruent; L = left; MFG = middle frontal gyrus; MTG/STG = middle/superior temporal gyrus; OFC = orbitofrontal cortex; pSTS = posterior superior temporal sulcus; pre-SMA = pre-supplementary motor area; R = right.

The main effect of modality (Table 3; Supplemental Fig. S2) was significant within the right anterior STG extending into the right parahippocampal gyrus (0 > attend-visual > attend-auditory) and the left secondary visual cortex (attend-visual > attend-auditory > 0).

**Table 3:** Main effect of modality during probe phase of the multisensory cognitive control task.

Area	BAs	CoM (X,Y,Z)	Volume	Pattern
Right anterior STG/parahippocampal gyrus	13/34/38/47	33, 8, −12	658 μL	AA > AV (deactivation)
Left secondary visual cortex	18	−24, −89, −7	3067 μL	AV > AA

*Note*: AA = attend-auditory; AV = attend-visual; BAs = Brodmann areas; CoM = Center of Mass; STG = superior temporal gyrus.

**Fig. 4:**
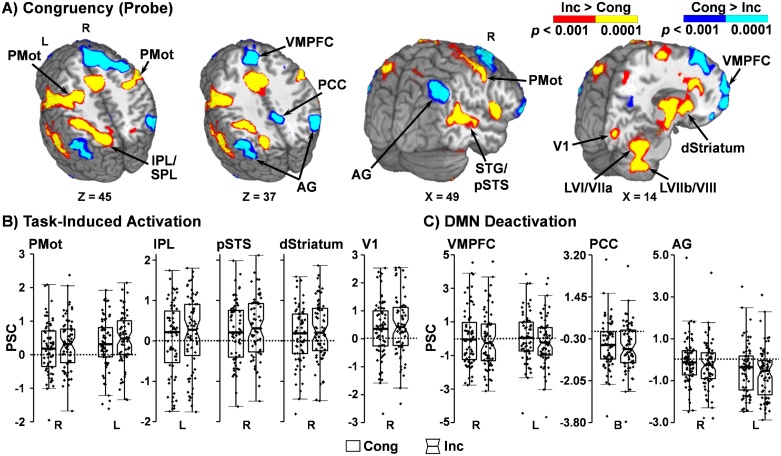
Main effect of congruency and percent signal change (PSC) during probe-related activity in selected regions of interest (ROI). Panel A presents regions that exhibited significant main effects of congruency during the multisensory task. Locations of the sagittal (X) and axial (Z) slices are given according to the Talairach atlas. Panels B displays box-and-scatter plots of the PSC from in selected ROIs (right = R; left = L; bilateral = B) showing increased activation for incongruent (Inc: notched boxes) relative to congruent (Cong: un-notched boxes) trials (Panel A warm colors: red p < 0.001; yellow p < 0.0001) within the premotor cortex (PMot), inferior/superior parietal lobule (IPL/SPL), superior temporal gyrus (STG) and posterior STS (pSTS), dorsal striatum (dStriatum), primary visual cortex (V1), and cerebellar Lobules VI/VIIa (LVI/VIIa) and VIIb/VIII (LVIIb/VIII). Ventromedial prefrontal cortex (VMPFC), posterior cingulate cortex (PCC), and angular gyrus (AG) showed greater deactivation (Panel A cold colors: blue p < 0.001; cyan p < 0.0001) during incongruent trials, and are depicted in Panel C with box-and-scatter plots.

Increased activation was observed on incongruent relative to congruent trials (Fig. 4A and B) within the right and left premotor cortex, right pSTS, left inferior/superior parietal lobule, right primary visual cortex, right and left dorsal striatum, brainstem extending into the cerebellar peduncles, bilateral cerebellar lobules I–VI and the vermis of lobule VI, right and left lobules VI/VIIa, and right and left lobules VIIb/VIIIa/VIIIb. A separate cluster of increased activation during the congruent relative to incongruent condition was also observed in left lobule VIIa of the cerebellum. Further, several areas including the default mode network presented a main effect of congruency, with increased deactivation in the incongruent relative to congruent condition (Table 4; Fig. 4A and C). These areas included the right and left ventromedial PFC and superior PFC extending to the right DLPFC, right orbitofrontal cortex, right MTG, bilateral posterior cingulate cortex, right cuneus/precuneus, and the right and left angular gyrus. In contrast, the right superior parietal lobule exhibited increased deactivation for congruent relative to incongruent trials

**Table 4:** Main effect of congruency during probe phase of the multisensory cognitive control task.

Area	BAs	CoM (X,Y,Z)	Volume	Pattern
**Task Induced Activations**				
Bilateral premotor cortex	R: 6	R: 33, −14, 53	R: 9488 μL	I > C
	L: 6	L: −27, −12, 54	L: 14120 μl	
Right posterior superior temporal sulcus	22/40	52, −40, 11	8998 μl	
Left inferior parietal lobule	40	−37, −39, 41	4912 μL	
Left inferior/superior parietal lobule	7/40	−23, −60, 45	11587 μl	
Right primary visual cortex	17	14, −85, 0	779 μL	
Bilateral dorsal striatum	–	R: 19, 6, 9	R: 7691 μL	
		L: −17, 4, 9	L: 6961 μL	
Bilateral brainstem and cerebellar peduncles	–	1, −24, −15	3745 μL	
Bilateral lobules I–VI of cerebellum and vermis of lobule VI	–	1, −53, −18	14954 μL	
Bilateral lobules VI/VIIa of cerebellum	–	R: 27, −51, −25	R: 9395 μL	
		L: −24, −48, −25	L: 5176 μL	
Bilateral lobules VIIb/VIIIa/VIIIb of cerebellum	–	R: 21, −55, −41	R: 8527 μL	
		L: −25, −48, −42	L: 4843 μL	
Left lobule VIIa of cerebellum	–	−39, −68, −38	1477 μL	C > I
**Default Mode Network & Other Task Induced Deactivations**				
Bilateral VMPFC/superior PFC and right DLPFC	R1: 10/11	R1: 24, 56, 3	R1:10207 μL	C > I (deactivation)
	L1: 10/11	L1: −22, 58, 4	L1: 838 μL	
	R2: 8/9	R2: 17, 27, 43	R2: 15807 μL	
	L2: 8	L2: −25, 21, 52	L2: 1863 μL	
Right orbitofrontal cortex	11/32	9, 37, −10	786 μL	
Right middle temporal gyrus	21	60, −16, −17	1620 μL	
Bilateral posterior cingulate cortex	31	4, −42, 37	1482 μL	
Right cuneus/precuneus	7/19/31	11, −65, 29	596 μL	
Bilateral angular gyrus	R: 7/39/40	R: 44, −62, 37	R: 3942 μL	
	L: 39/40	L: −44, −63, 35	L: 2851 μL	
Right superior parietal lobule	7	18, −64, 51	980 μL	I > C (deactivation)

*Note*: BAs = Brodmann areas; C = congruent**;** CoM = Center of Mass; DLPFC = dorsolateral prefrontal cortex; I = incongruent; L = left; PFC = prefrontal cortex; R = right; VMPFC = ventromedial prefrontal cortex.

### 3.3 Connectivity analysis

The connectivity analysis (N = 66) examined whether certain voxelwise regions of the brain would demonstrate a resting-state connectivity bias with atlas-derived primary/secondary auditory or visual seed ROI, consistent with the modality-specific biases (attend-visual > attend-auditory or attend-auditory > attend-visual) that were observed during the probe phase of the multisensory task. Specifically, paired-samples t-tests evaluated whether whole-brain voxelwise connectivity was greater with auditory or visual atlas seeds (See Supplementary Fig. S1). Regions that exhibited both significant auditory or visual seed functional connectivity bias and a significant effect of modality from task analyses were determined by implementing an additional small-volume overlap correction (2 native voxels; 54 μL) on both modality (i.e., task-based) and connectivity maps.

Results indicated that both areas showing an effect of modality during probe analysis, the right anterior STG extending into the right parahippocampal gyrus (BAs 13/34/38/47; 626 μL) and left secondary visual cortex (BAs 17/18; 1207 μL), overlapped significantly with areas displaying connectivity differences between unisensory cortices. Of particular note, these results were complementary (i.e., greater visual seed connectivity where activation was greater to attend-auditory stimuli, greater auditory seed connectivity where activation was greater to attend-visual stimuli).

In addition to overlapping areas, the connectivity analysis showed widespread differences in connectivity between unisensory seeds (Supplementary Fig. S1). The pre-motor cortex and much of the posterior parietal lobe, occipital lobe, and cerebellum displayed greater connectivity to the visual cortex seed than to the auditory cortex seed. Conversely, the dorsomedial PFC and the dorsal anterior/middle cingulate gyrus, the insula, and a large portion of the temporal lobe displayed greater auditory cortex seed connectivity. These results were replicated when using unisensory cortex seeds derived from ARMs contrast maps rather than atlas-based seeds (see Supplemental Fig. S1).

## 4 Discussion

The current study theoretically examined proactive and reactive multisensory cognitive control in adolescents by adapting a previously used task to disambiguate cue and probe-related activity (Mayer et al., 2017). Contrary to our hypothesis and previous work in adults (Braga et al., 2013, 2017; Mayer et al., 2017; Michalka et al., 2015; Noyce et al., 2017), modality-specific stratification of the PFC was not observed when directing attention to either visual or auditory stimuli during multisensory presentation. Instead, several of the CCN nodes (left DLPFC, bilateral pre-SMA, bilateral anterior insular cortex/ventrolateral PFC, bilateral ACC) and the left STG/pSTS exhibited modality-specific activation for incongruent relative to congruent trials during attend-visual trials only. The remainder of the CCN and the right pSTS demonstrated increased activity during incongruent trials as predicted. Finally, ARMs were observed within the visual cortex during reactive cognitive control following probes, but were absent within unisensory cortex during more proactive phases of cognitive control following cues.

As expected, response times were faster for congruent relative to incongruent trials, along with robust activation of several traditional nodes of the CCN including posterior parietal cortex and dorsal striatum, as well as the cerebellum (Braver et al., 2009; Wu et al., 2020a). Although medial and lateral PFC were further modulated by the modality for focused attention, these results suggest that the reactive CCN is fully developed in adolescents (Gonthier et al., 2019). Previous findings (Mayer et al., 2017) of increased involvement of the pSTS during multisensory reactive cognitive control in an adult sample were also replicated in adolescents. The pSTS is located between the auditory and ventral visual streams, and traditionally has been associated with the integration of information across these two sensory modalities (Deen et al., 2015; Beauchamp et al., 2004). However, differentiation of activation based on stimulus characteristics (e.g., congruent vs. incongruent) suggests a higher-order attentional role for the pSTS during multisensory processing.

As previously described, recent studies have suggested that the PFC is activated in both a supramodal (Wu et al., 2020a,b) or modality-specific (Mayer et al., 2017; Michalka et al., 2015; Noyce et al., 2017; Braga et al., 2013, 2017) fashion. In contrast, the PFC in the current adolescent sample did not exhibit evidence of stratification based on sensory modality during either the theoretical proactive or reactive multisensory cognitive control aspects of the task. Similarly, connectivity analyses did not provide support for either a rostral lateral PFC connectivity with the auditory cortex (Mayer et al., 2017) or a dorsal-to-ventral PFC gradient with unisensory cortex (Braga et al., 2017; Michalka et al., 2015). Instead, current findings indicated that the lateral (left DLPFC, ventrolateral PFC) and medial (pre-SMA/ACC) PFC showed greater evidence of reactive control only during the attend-visual condition (Modality × Congruency interaction).

The interaction in prefrontal cortical areas may be explained by more protracted neurodevelopmental changes that occur in the neural recruitment of the PFC (Crone and Steinbeis, 2017; Luna et al., 2015), and potentially reflects a continued transitional bias away from favoring auditory stimuli from earlier developmental periods. Behavioral performance was also mixed in terms of sensory dominance. Specifically, adolescents displayed a greater bias toward visual relative to auditory stimuli during reactive cognitive control as evidenced by faster overall reaction times and increased accuracy on congruent trials. Higher accuracy was observed for incongruent attend-auditory trials, suggesting a strong influence of auditory distractors while processing visual probes. Collectively, current behavioral and functional findings do not fully support the consolidation of visual dominance in adolescents aged 13–18 years old during multisensory processing (Barnhart et al., 2018; Chen et al., 2016; Hirst et al., 2018a,b; Nava and Pavani, 2013; Robinson and Sloutsky, 2004; Wille and Ebersbach, 2016).

Discrepancies in PFC stratification between current and past findings might also be due to methodological differences in task design. Specifically, the interval between the start of the cue and start of probes was intentionally increased (greater than 2500 ms) and made to be a variable rather than fixed interval (1000 ms) as used in previous studies (Mayer et al., 2017). This change in experimental design permitted the individual modeling of HRFs for cues and probes, which theoretically disambiguates the neural substrates of proactive (cue-related activity) and reactive (probe-related activity) control. However, neither stratification of the PFC nor anticipatory modulation of unisensory cortex (ARMs) was observed during the cue phase. Instead, increased activation was observed within the right DLPFC, temporal pole, MTG/STG and precuneus following visual relative to auditory cues, along with increased deactivation of the anterior default mode network during the attend-auditory trials for periods in which the HRF is typically associated with inhibition (Mayer et al., 2020).

Importantly, previous research also suggests that different experimental manipulations can contribute to the weighting of proactive versus reactive control processes (Braver et al., 2009; Gonthier et al., 2016; van Wouwe et al., 2009). For example, shortening the delay between cue and probe reduces age-related differences in performance on the AX-CPT task (Lorsbach and Reimer, 2010). As such, longer inter-stimulus intervals utilized in the current experiment may have also influenced how individuals utilize cue information and the recruitment of the lateral PFC. Moreover, the cue did not provide any information about upcoming probe status (i.e., congruent or incongruent stimuli), which may have limited its overall utility and further reduced proactive use. In addition, the current design did not collect reaction time data under differential conditions to verify that participants were in fact proactively using the cues, as is typically done with the AX-CPT and other control tasks (Gonthier et al., 2019; Lorsbach and Reimer, 2010; Polizzotto et al., 2018; Ryman et al., 2019).

Similar to previous findings (Mayer et al., 2017), ARMs were more evident in the visual cortex in the analysis of probes, showing evidence of both up-regulation during attend-visual trials (i.e., left secondary visual cortex) and increased deactivation during attend-auditory trials (i.e., left associative visual cortex). Increased deactivation of the left STG/pSTS was present during the attend-visual relative to attend-auditory trials, providing only minimal evidence of attentional modulations within auditory cortex. Both the unilateral presentation (left hemisphere only) and overall reduction in volume for visual and auditory cortical ARMs was unexpected in our adolescent sample. Previous studies have demonstrated differences in value-driven attentional capture (Roper et al., 2014) and facial processing (Monk et al., 2003) between adolescents and adults, with the latter study indicating increased modulation of task-relevant networks based on attentional demands for adults relative to greater emotional modulation for adolescents. Collectively, current and previous findings suggest that higher level attentional capabilities are not fully developed in adolescents.

Several limitations of the current study should be noted. An older adolescent age group was included (13–18 years old), which precluded the study of earlier neurodevelopmental aspects of multisensory attention and cognitive control. Therefore, it is unclear whether current findings would apply to younger children, especially in response to cue-related activity as children tend to behave/respond more reactively (Gonthier et al., 2019; Munakata et al., 2012). Future studies are needed to more fully explore developmental proactive-to-reactive shifts and sensory dominance across childhood to adulthood. Second, as previously discussed, the use of a different version of the multisensory task precludes disambiguation of whether discrepant results are due to variations in tasks used or secondary to developmental effects.

Collectively, current behavioral and functional findings suggest a continued rather than a completed development towards visual dominance during adolescence. Modality-specific stratifications of the lateral PFC were also not observed, with only moderate evidence of ARMs in unisensory cortical regions. Given that this study is the first to investigate brain activation during multisensory cognitive control in adolescents, these findings will need to be replicated in independent samples and across the full neurodevelopmental spectrum.

## Data statement

The data that support the findings of this study will be openly available in FITBIR at fitbir.nih.gov upon the conclusion of the study, reference number FITBIR-STUDY0000339.

## CRediT authorship contribution statement

**V. Sicard:** Methodology, Formal analysis, Writing - original draft, Visualization. **D.D. Stephenson:** Methodology, Formal analysis, Writing - original draft. **A.B. Dodd:** Software, Validation, Formal analysis, Visualization, Writing - review & editing. **S. Pabbathi Reddy:** Investigation, Data curation, Writing - review & editing. **C.R. Robertson-Benta:** Investigation, Data curation, Writing - review & editing. **S.G. Ryman:** Writing - review & editing. **F.M. Hanlon:** Writing - review & editing. **N.A. Shaff:** Software, Formal analysis. **J.M. Ling:** Software, Data curation. **D.C. Hergert:** Investigation, Writing - review & editing. **K. Vakamudi:** Writing - review & editing. **J. Hogeveen:** Writing - review & editing. **A.R. Mayer:** Conseptualization, Methodology, Resources, Writing - review & editing, Supervision, Funding acquisition.

## Declaration of Competing Interest

The authors report no declarations of interest.
