# Supplementary material for: Is the prefrontal cortex organized by supramodal or modality-specific sensory demands during adolescence?
Source: Dev Cogn Neurosci. 2021 Aug 14;51:101006. doi: 10.1016/j.dcn.2021.101006 (PMC8379626; doi:10.1016/j.dcn.2021.101006)
Supplement: Supplementary file 1 [file mmc1.docx]

**Supplementary Material for ‘Is the prefrontal cortex organized by supramodal or modality-specific sensory demands during adolescence?’**

1. **MR Imaging Parameters**

All participants were scanned on a 3T Siemens Trio system with a 32-channel head coil. Foam padding was used to minimize head motion. A high resolution 5-echo Magnetization Prepared Rapid Acquisition Gradient Echo (MPRAGE) T_1_–weighted [repetition time (TR)=2530 ms; echo times (TE)=1.64, 3.50, 5.36, 7.22, 9.08 ms; inversion time (TI)=1200 ms; flip angle=7°; number of excitations (NEX)=1; slice thickness=1 mm; field of view (FOV)=256 mm; matrix size=256 × 256; isotropic voxels=1 mm] was collected.

Resting state data was acquired over a 5-minute period using a single-shot, gradient-echo echoplanar pulse sequence [TR=460 ms; TE=29 ms; flip angle=44°; multiband acceleration factor=8; NEX=1; slice thickness=3 mm; FOV=248 mm; matrix size=82 × 82] with 56 interleaved 3 mm slices acquired for whole-brain coverage (voxel size=3.02 × 3.02 × 3.00 mm). Due to increased grey-white contrast, a reference image with multiband acceleration factor set to one (i.e., no acceleration) was acquired to facilitate registration with the native T_1_-weighted anatomical image.

Finally, two spin-echo field mapping sequences [TR=7220 ms; TE=73 ms; flip angle=90°; refocus flip angle=180°; slice thickness=3 mm; FOV=248 mm; matrix size=82 × 82; 56 interleaved slices; voxel size=3.02 × 3.02 × 3.00 mm] with reversed phase encoding directions (A→P; P→A) collected to account for susceptibility distortions in the gradient echo data.

1. **Connectivity Analysis**

Connectivity analyses aimed to examine whether regions that exhibited modality-specific effects during the multisensory task would show a similar directional bias in terms of resting-state functional connectivity with seeds from either the auditory or visual cortex.

The seeds for primary/secondary auditory or visual cortices were defined through anatomical labels from FreeSurfer (version 5.3) using the Desikan-Killiany atlas (Desikan et al., 2006). Primary (Heschl’s gyrus; A1) and secondary (planum temporal, Heschl’s sulcus, planum polare, and superior temporal gyrus; A2) auditory cortex were defined using previously published labels (Destrieux, Fischl, Dale, & Halgren, 2010). Secondary visual cortex (V2) was identified using Fischl’s label (Fischl et al., 2008), whereas primary visual cortex (V1) was defined based on the Hinds’ label (Hinds et al., 2008) with areas shared by the V2 label removed.

In addition to the anatomically-derived seed-based analysis presented in the main text, we also derived empirical seeds using those unisensory cortical regions that exhibited high activation in response to the relevant auditory and visual probes. Again, paired-sample t-test evaluated whether connectivity was greater for auditory or visual seeds. Regions indicating both significant functional connectivity and main effect of modality during the probe analysis were determined by conducting an additional small-volume overlap correction (2 native voxels; 54 μL), imposed on the individually corrected results from both the main effect of modality and the connectivity results. The thresholding criteria were based on 10,000 Monte Carlo simulations based on the main effect of modality, using a family-wise error threshold of *p*< 0.001. Comparison of significant connectivity between auditory and visual cortex using either empirically-derived or anatomically-derived seeds are presented in Figure S1. The pattern of results was largely similar across both methods.

1. **Functional Analyses with Age as a Covariate**

**Functional analyses for both the cue and probe phases of the task were repeated with the inclusion of age as an additional covariate. Cue analyses of both the peak and inhibitory phases were similar, with no changes in identified clusters. Clusters in both main effects and the interaction from the probe phase analysis were also similar. The only exception was for the main effect of Congruency, with the right cuneus/precuneus (previously 596 µL) no longer significant due to falling below the minimum required cluster size threshold (575 µL).**

**References**

Desikan, R. S., Segonne, F., Fischl, B., Quinn, B. T., Dickerson, B. C., Blacker, D. et al. (2006). An automated labeling system for subdividing the human cerebral cortex on MRI scans into gyral based regions of interest. *Neuroimage., 31*(3), 968-980. Retrieved from PM:16530430

Destrieux, C., Fischl, B., Dale, A., & Halgren, E. (2010). Automatic parcellation of human cortical gyri and sulci using standard anatomical nomenclature. *Neuroimage., 53*(1), 1-15.

Fischl, B., Rajendran, N., Busa, E., Augustinack, J., Hinds, O., Yeo, B. T. et al. (2008). Cortical folding patterns and predicting cytoarchitecture. *Cereb.Cortex, 18*(8), 1973-1980. doi:bhm225 [pii];10.1093/cercor/bhm225 [doi]. Retrieved from PM:18079129

Hinds, O. P., Rajendran, N., Polimeni, J. R., Augustinack, J. C., Wiggins, G., Wald, L. L. et al. (2008). Accurate prediction of V1 location from cortical folds in a surface coordinate system. *Neuroimage., 39*(4), 1585-1599. doi:S1053-8119(07)00957-3 [pii];10.1016/j.neuroimage.2007.10.033 [doi]. Retrieved from PM:18055222


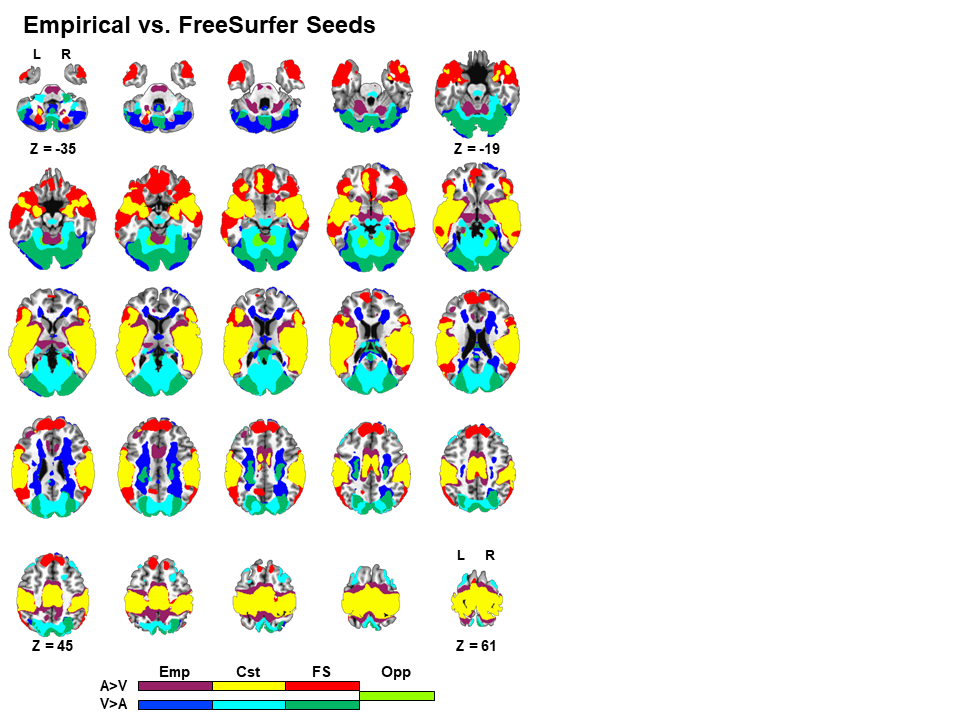


**Supplementary Figure S1**. Results comparing significant (minimum p<0.001 and minimum cluster size = 54 μL) **functional** connectivity between auditory and visual cortex where seeds were empirically-derived relative to using FreeSurfer (FS) labels for seeds. Regions exhibiting increased connectivity for auditory (A) relative to visual (V) cortex are represented in warm colors, with yellow indicating regions that showed consistent (Cst) patterns of connectivity across both methods. Purple (empirically-derived seeds; Emp) and red (FS) colors indicate method-specific differences in connectivity. Regions exhibiting increased connectivity for visual relative to auditory cortex are represented in cool colors (Emp = blue; FS = green; consistent across both methods = cyan). Select axial slices are displayed at 4 mm intervals according to the Talairach atlas with the right (R) and the left (L) hemispheres denoted. The pattern of results was generally similar across both methods, with the empirically-derived seeds typically resulting in larger volumes of connectivity relative to FS labels. However, differential patterns of activation were observed in frontal regions. Few cortical regions exhibited opposite **functional connectivity** results (Opp = chartreuse) dependent on whether seeds were empirically derived or based on anatomical labels.

**
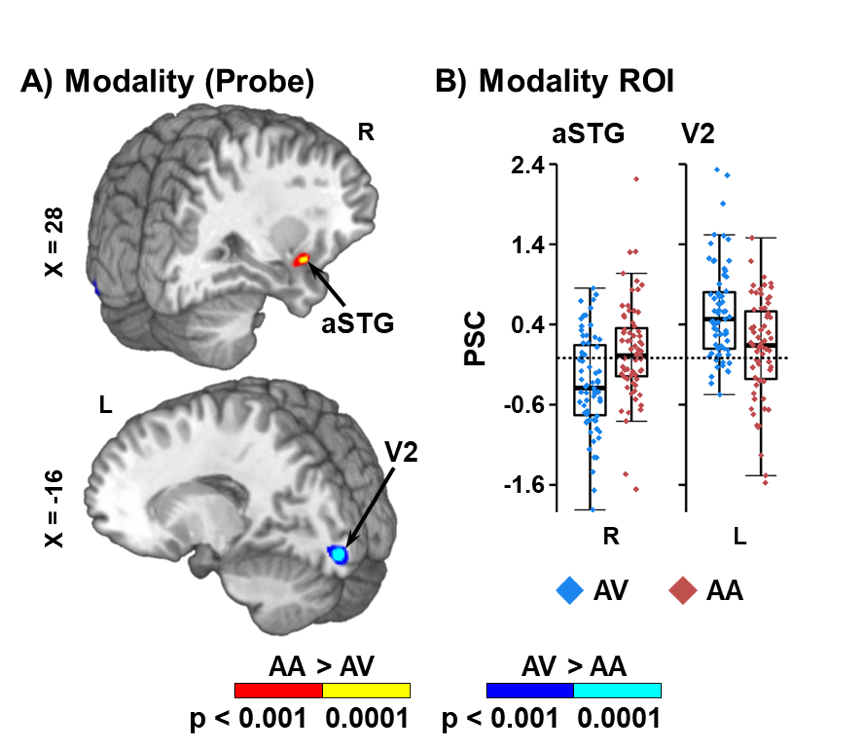
**

**Supplementary Figure S2**. Probe phase main effect of modality and percent signal change (PSC). Greater activation during attend-auditory (AA) trials (red: p<0.001; yellow: p<0.0001) was observed within the right (R) anterior superior temporal gyrus (aSTG), whereas greater activation during attend-visual (AV) trials (blue: p<0.001; cyan: p<0.0001) was observed within the left (L) secondary visual cortex (V2). Location of sagittal (X) slices are given according to the Talairach atlas. Panel B displays box-and-scatter plots of the PSC in these regions (blue: AV; red; AA).
